# Purinoceptor Targeted Cytotoxicity of Adenosine Triphosphate-Conjugated Biogenic Selenium Nanoparticles in Human Colon Cancer Cells

**DOI:** 10.3390/ph15050582

**Published:** 2022-05-06

**Authors:** Kandasamy Saravanakumar, Anbazhagan Sathiyaseelan, Xin Zhang, Soyoung Park, Myeong-Hyeon Wang

**Affiliations:** Department of Bio-Health Convergence, Kangwon National University, Chuncheon 200-701, Korea; saravana732@gmail.com (K.S.); sathiyaseelan.bio@gmail.com (A.S.); zhangxin199708@gmail.com (X.Z.); dsj10140@naver.com (S.P.)

**Keywords:** selenium, caco-2 cell line, anticancer, adenosine triphosphate

## Abstract

The adenosine triphosphate (ATP)-conjugated biogenic selenium nanoparticles (SeNPs) for P2 (purinoceptors) receptor-targeted anti-colon cancer activity were developed in this study. First, the SeNPs were synthesized using *Trichoderma* extracts (TE) and then conjugated with ATP to enhance their anticancer activity. The developed SeNPs had an oval crystalline structure with an average diameter size of 26.45 ± 1.71 d. nm, while the ATP-SeNPs were 78.6 ± 2.91 d. nm. The SeNPs contain Se, and less persistence of P while the ATP-SeNPs have high level of P, and Se in the energy-dispersive spectroscopy (EDS). Further, both nanoparticles exhibited larger sizes in the dynamic light scattering (DLS) analysis than in the transmission electron microscopy (TEM) analysis. The DLS and Fourier transform infrared spectroscopy (FTIR) results provide evidence that the amine group (–NH_2_) of ATP might bind with the negatively charged SeNPs through covalent bonding. The IC_50_ concentration was 17.25 ± 1.16 µg/mL for ATP-SeNPs and 61.24 ± 2.08 µg/mL against the caco-2 cell line. The IC_50_ results evidenced the higher cytotoxicity of ATP-SeNPs in the caco-2 cell line than in HEK293 cells. ATP-SeNPs trigger the anticancer activity in the caco-2 cell line through the induction of mitochondrial membrane potential (MMP) loss and nucleus damage. The biocompatibility test of hemolysis and the egg CAM assay confirmed the non-toxicity of these nanoparticles. Overall, the results proved that the newly developed ATP-SeNPs exhibited higher cytotoxicity in the caco-2 cell line than SeNPs. However, further molecular and in vivo experiments are required to develop the ATP-SeNPs as a candidate drug for cancer-targeted therapeutics.

## 1. Introduction

Colorectal cancer (CRC) is the third most prevalent cancer that affects human health worldwide, and the mortality rate is expected to be 60% in the year 2030 [1,2]. The current treatment and diagnosis methods are inefficient for the successful detection and treatment of CRC, which requires the development of advanced methods for improved diagnosis and therapy specifically for CRC. In this contest, nanomedicine is a convergence of biology, materials engineering, medicine, and technology, which presents an attractive strategy for a diverse array of applications in disease diagnosis and therapeutics [3]. These nanoparticles are found to be promising in diverse biological applications, compared to bulk solids or molecules, due to their unique structural, radiological, magnetic, and optical properties, which lead to their utilization in cancer diagnosis, cancer imaging, and targeted therapy [4,5]. Nanomedicine has high potential compared to conventional cancer therapeutics, such as surgical or basic radiation therapy, because the ligand-functionalized nanomaterials target the pathological site without causing any adverse effects [6]. Nanomedicine presents attractive inorganic and organic nanoparticles for cancer diagnosis and therapeutics. Among nanoparticles, SeNPs have distinct properties such as biocompatibility, bioavailability, bio-efficacy, antioxidant activity, and disease prevention [7,8].

Selenium is recognized as an essential trace element and its supplementation can provide several health benefits for humans [9]. In particular, selenium supplementation is known to reduce the risk of cancers, including colon, lung, and liver cancers [10,11]. The anticancer activities of selenium have been influenced by its dose, metabolic activities, and chemical form [12,13,14]. The chemical form of selenium compounds, including methyl selenocysteine, methylseleninic acid, and selenomethionine, poses excellent therapeutic effects on liver cancers [15]. The nano-size and the lower surface area–volume ratio of SeNPs result in less toxicity and excellent bioavailability compared to selenium salts [16]. Thus, the SeNPs are considered to be a promising candidate for cancer therapeutics compared to the selenium compounds, including selenate (Se^II^) and selenite (Se^IV^), due to their low bioavailability [17]. Therefore, SeNPs are gaining attention in nano-biomedicine as potentially effective anticancer agents with low toxicity [18]. Although SeNPs exhibit potential anticancer activities, their poor cellular uptake limits their clinical applications [19]. Therefore, the ligand conjugation can increase the cellular uptake of SeNPs, resulting in higher cytotoxicity. For example, ATP-conjugated selenium nanoparticles show potential anticancer activity in human hepatocellular carcinoma cells (HepG2 cells) by targeting the P2 [3]. ATP is known to recognize the purinoceptors present in various cancer cells, including human colon cancer cells (caco-2 cells) [20,21]. In addition, it is reported that ATP can act as a pro-drug for the suppression of caco-2 cell proliferation via protein kinase C (PKC) inhibition, as well as assist in internalizing biological macromolecules through the P2 [22,23].

The nanomaterials are prepared by various physicochemical and biological methods, but among them, the biological methods are considered to be eco-friendly and are preferred over the chemical methods [24]. The synthesis of nanoparticles using microorganisms, algae, and plants is a reliable approach that does not produce toxic substances, is economically viable, and is ecologically friendly [25,26]. Among the microorganisms, probiotic bacterial strains are known to resist the toxicity of selenium through biological conversion of the selenium oxyanions to less toxic selenium (0) nanoparticles through the detoxification or redox homeostasis mechanism [27,28,29,30]. *Trichoderma* extracts are known to contain several proteins, volatile and nonvolatile metabolites, and peptides [31]. Moreover, *Trichoderma* sp. synthesizes SeNPs with inhibitory activity against the plant pathogen *Sclerospora graminicola* [31]. However, there are no reports of anticancer activity of *Trichoderma* sp. extracts-mediated SeNPs. Therefore, this study aimed to synthesize and characterize SeNPs using the native fungus *T. harzianum* (SKCGW012), then conjugate ATP to the surface of the SeNPs so that they could be used to kill human colon cancer cells (caco-2 cells) through enhanced cellular uptake and cell death.

## 2. Results and Discussion

### 2.1. Synthesis and Characterization of Nanoparticles

Secretomes including volatile and nonvolatile compounds, metabolites, proteins, and peptides in the TE were involved as reducing or capping agents in the synthesis of SeNPs. The synthesis of SeNPs through the interaction of the TE and sodium selenite was visualized by color changes followed by characterized of applying the following methods: UV–vis spectrophotometer, XRD, DLS, TEM, EDS, and FTIR. 

#### 2.1.1. UV–Vis Spectrophotometer Analysis

**Figure 1:**
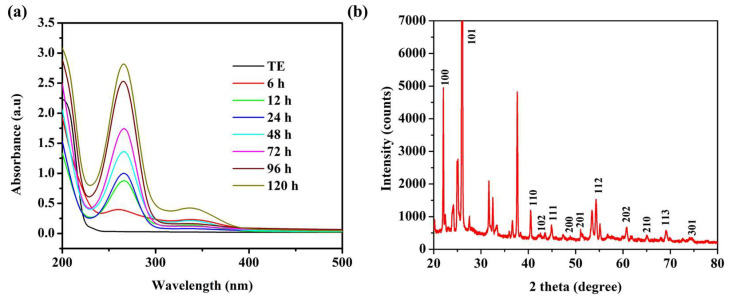
UV–Vis spectrophotometer analysis (**a**), and X-ray diffraction (XRD) pattern analysis (**b**) of selenium nanoparticles (SeNPs); TE—*Trichoderma* extract.

The SeNPs were synthesized using TE and were conjugated with ATP for enhanced cytotoxicity in the caco-2 cell line. The reaction mixture color changed from yellow to ruby red and the strong absorption peak ranged from 250–290 nm with a λ max of 270 nm observed in the UV–Vis spectrophotometer analysis, revealing the successful formation of SeNPs (Figure 1a). In addition, it was noted that the λ max was significantly increased with the increase in the reaction period. Similarly, an earlier study demonstrated that SeNPs exhibit a λ max of 259 nm and 252 nm for *Trichoderma* culture extract- and plant extract-mediated synthesis of SeNPs, respectively [32,33]. The effective formation of the SeNPs is reported in a slightly alkaline condition, and the morphology and size of the nanoparticles are studied by the UV–Vis spectrophotometry. It has been reported that absorption peaks ranging from 200–400 nm reveal the formation of surface plasmon resonance (SPR) of SeNPs with a mean size of 120 nm [34,35].

#### 2.1.2. X-ray Diffraction (XRD) Pattern Analysis

Selenium (Se) can occur in three phases: triclinic-Se, monoclinic-Se, and amorphous. An XRD analysis was performed to determine the crystalline characteristics of SeNPs. The SeNPs exhibited the 2θ planes at 100, 101, 110, 102, 111, 200, 201, 112, 202, 210, 113, and 301, corresponding to SeNPs (Figure 1b). This result is matched with the Joint Committee on Powder Diffraction Standards (JCPDS file no. 06-0362) [36,37], which indicated the TE-mediated formation of oval crystalline SeNPs. These results agree with the earlier reports of the *Trichoderma* sp. extracts-mediated synthesis of SeNPs [32,38]. The unidentified peaks in the present XRD spectrum corresponded to the capping or coating of the bioactive compounds of TE (Figure 1b). In addition, the same kind of noise peaks has been reported in the past studies of SeNPs synthesis with *Trichoderma* sp. and Fenugreek seed extracts [38,39].

#### 2.1.3. Morphological Characteristics

**Figure 2:**
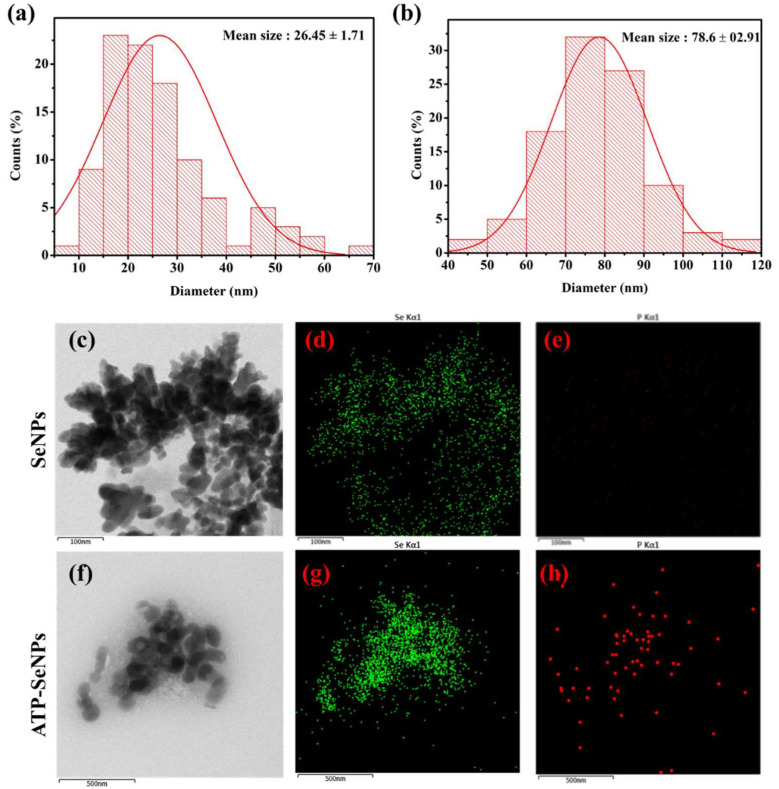
Particle size distribution analysis of SeNPs (**a**) and adenosine triphosphate-conjugated SeNPs (ATP-SeNPs; (**b**), transmission electron microscopic (TEM) and energy-dispersive X-ray spectroscopy (EDS) analysis of SeNPs (**c**–**e**) and ATP-SeNPs (**f**–**h**). The EDS analysis showed mapping of selenium (**d**,**g**) and phosphate (**e**,**h**) in SeNPs and ATP-SeNPs, respectively.

**Figure 3:**
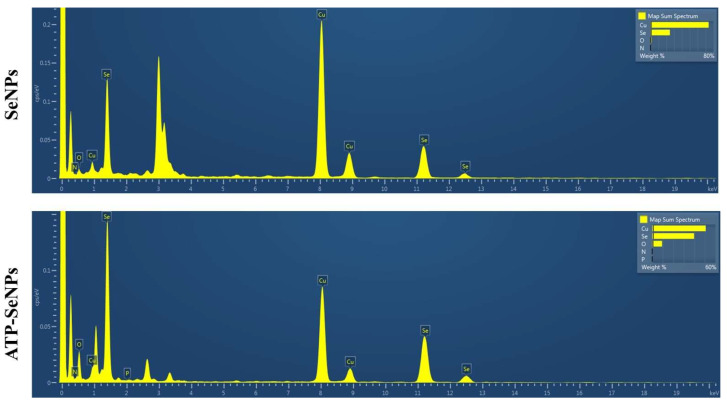
Energy-dispersive X-ray spectroscopy (EDS) analysis of SeNPs and ATP-SeNPs. The appearance of Se and P in the ATP-SeNPs indicates the successful fabrication of ATP-conjugated SeNPs.

The size and morphological properties play a vital role in the bioactive function of the nanoparticles, including antioxidant activity, antibacterial enzyme induction, and cytotoxicity [40]. Thus, the size, morphology, and elemental composition of the SeNPs and ATP-SeNPs were observed by TEM-EDS analysis (Figure 2). Both nanoparticles were found to be monodispersed and oval structures with an average diameter size of 26.45 ± 1.71 d. nm for SeNPs and 78.6 ± 2.91 d. nm for ATP–SeNPs (Figure 2a,b). The SeNPs were aggregated and exhibited the elemental composition of Se in the less persistence of phosphate (P), which is evidenced by EDS mapping (Figure 2c,e). The high aggregation of both nanoparticles occurred due to the high surface energy, which is in agreement with earlier work [3]. The TEM image displayed the aggregated ATP–SeNPs with a thin membrane layer covering, which might be because of ATP conjugation. In the earlier work, the thin membrane-like coating on the SeNPs was reported due to the encapsulation of the tuna and tilapia polypeptides on SeNPs [41,42]. In addition, the elemental analysis resulted in the presence of Se, O, and P atoms in the ATP–SeNPs (Figure 2f–h), which indicated the successful conjugation of ATP in SeNPs. These results were further evidenced by the EDS chromatogram (Figure 3). Furthermore, an earlier study demonstrated the successful conjugation of ATP on SeNPs by elemental analysis using EDS [3].

#### 2.1.4. Hydrodynamic Size and Zeta Potential

**Figure 4:**
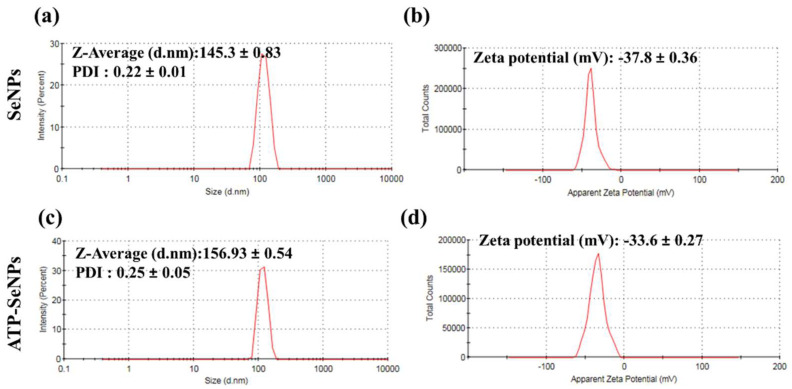
Dynamic light scattering (DLS) analysis of SeNPs and ATP-SeNPs. Zeta size and potential of SeNPs (**a**,**b**) and ATP-SeNPs (**c**,**d**).

The average size and zeta potential of SeNPs and ATP-SeNPs were measured using the zeta size analyzer (Figure 4). The average diameter of the hydrodynamic size of the SeNPs was found to be 145.3 ± 0.83 d. nm with a polydispersity index (PDI) of 0.22 ± 0.01 and a zeta potential of −37.8 ± 0.36 mV (Figure 4a,b). In the case of ATP-SeNPs, the average diameter of the hydrodynamic size was 156.93 ± 0.54 nm with a PDI of 0.25 ± 0.05 and zeta potential of −36.6 ± 0.27 nm (Figure 4c,d). This result indicated that the amine group (-NH_2_) of ATP might bind with the negatively charged SeNPs through covalent bonding by electrostatic interaction while the PO_2_-coated on the surface of the ATP-SeNPs resulted in the negative charge of the ATP-SeNPs. This result is in agreement with the earlier work [3]. Moreover, the PDI of both nanoparticles indicated the mono dispersion nature with good stability that is amicable for biomedical applications. According to studies, a PDI < 0.5 and an average diameter size < 200 nm increase bioactivity by enhancing cellular penetration [6,43].

#### 2.1.5. Functional Group Analysis

**Figure 5:**
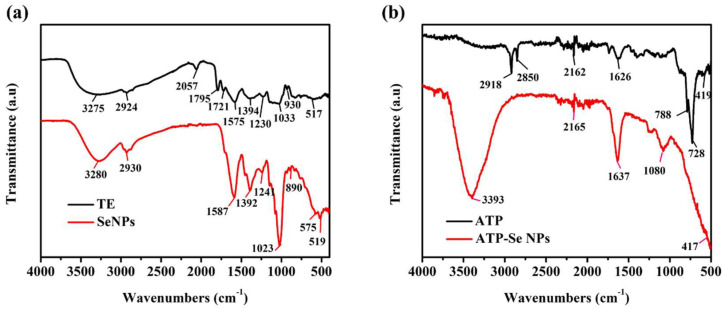
Fourier transform infrared spectroscopy (FTIR) analysis of *Trichoderma* sp extract (TE)-mediated SeNPs (**a**) and ATP-SeNPs (**b**).

The FTIR spectrum of TE exhibited bond vibrations at 3275 cm^−1^, 2924 cm^−1^, and 1394 cm^−1^ as characteristic vibrational bands of O–H stretching, 2057 cm^−1^ for C=C=C stretching, 1795 cm^−1^ and 1721 cm^−1^ characteristic vibrational bands of C=O stretching, 1575 cm^−1^ (C=C), 1230 cm^−1^, and 1033 cm^−1^ related to amine (C–N stretching), and 930 cm^−1^ for C–O (Figure 5a). The FTIR spectrum of SeNPs had the bond vibrations at 3280 cm^−1^ and 1392 cm^−1^ related to a hydroxyl group (O–H stretching), 2930 cm^−1^, 1587 cm^−1^, 1241 cm^−1^, and 1023 cm^−1^ corresponding to the characteristic vibrational bands of the amine group (N–H stretching and bending), and 890 cm^−1^ accounting for the alkene group (C=C bending). These FTIR spectrums of TE and SeNPs have indicated the capping of the protein and hydroxyl molecules from TE, which agrees with earlier works [38,44]. From the FTIR spectrum, it was seen that ATP exhibited the characteristic vibrational bands at 2918 cm^−1^, 2850 cm^−1^, and 1626 cm^−1^, corresponding to amine (N–H stretching), and 788 cm^−1^ and 728 cm^−1^, accounting for C=C bending [45]. The FTIR bond vibrations of ATP-SeNPs were found to be similar to ATP, and most of the bond vibrations were moved from ATP to ATP-SeNPs with slight shifting. For instance, vibrational bands such as 2165 cm^−1^, 1637 cm^−1^, and 417 cm^−1^ were found in the ATP-SeNPs with slight shifting. These findings confirm the successful conjugation of the ATP on the SeNPs. In addition, the disappearance of the vibrational bands such as 2918 cm^−1^ and 2850 cm^−1^ in the ATP, corresponding to the amine (N–H stretching), revealed the formation of ATP-SeNPs stable conjugation through the Se–NH conjugation bond [3].

### 2.2. Cytotoxicity

#### 2.2.1. WST-1 Assay and Cellular Uptake

**Figure 6:**
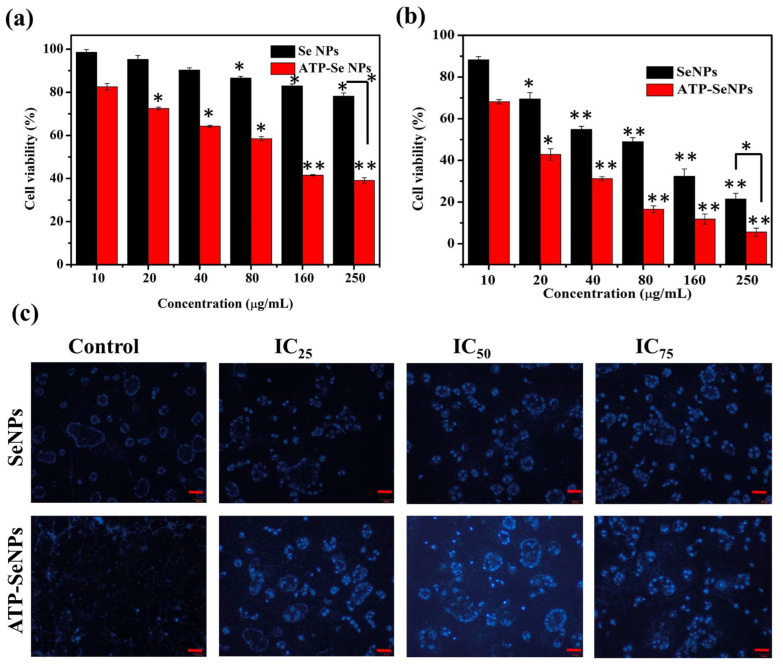
Cytotoxicity of SeNPs and ATP-SeNPs in HEK293 cell line and (**a**) human colon cancer (caco-2) cell lines, (**b**) and analysis of SeNPs and ATP-SeNPs-induced nucleus damage in caco-2 cells by DAPI staining assay (**c**). Scale bar (100 µm). * *p* < 0.05; ** *p* < 0.01 significantly varied between the low concentration (10 µg/mL) and between type of the nanoparticles.

Cytotoxicity of the SeNPs and ATP-SeNPs was tested in the two types of cell lines, the HEK293 cell line and the caco-2 cell line, by WST-1 assay (Figure 6a,b). The cytotoxicity of both nanoparticles was significantly increased with the increase in concentration (*p* < 0.05). The ATP-SeNPs exhibited higher cytotoxicity in caco-2 cell lines than HEK293 cell lines due to the purinoceptor targeting ability being enabled by ATP conjugation. The IC_50_ concentration was 17.25 ± 1.16 µg/mL for ATP-SeNPs and 61.24 ± 2.08 µg/mL against the caco-2 cell line (Figure 6a,b). In the case of the HEK293 cell line, the IC_50_ were 121.62 ± 1.04 µg/mL for ATP-SeNPs and 487.52 ± 2.95 µg/mL for SeNPs. The IC_50_ results evidenced the higher anticancer activity of ATP-SeNPs in the caco-2 cell line compared to HEK293 cells. Although the purinoceptor is found in all cells, it is found in higher expression in cancer cells such as hepatocellular carcinoma cells (HepG2 cell line) and human colon cancer cells (caco-2 cells) [3,20,21]. Moreover, the cellular uptake of nanoparticles was found to be higher with ATP-SeNPs (0.92 ± 0.02 µg/10^4^ cells) compared to SeNPs (0.24 ± 0.1 µg/10^4^ cells) due to the purinoceptor targeted penetration of the ATP-SeNPs. ATP acts as a pro-drug suppressor cell proliferation of caco-2 via protein kinase C (PKC) inhibition, which increases macromolecule internalization via the P2 purinoceptor [22,23].

#### 2.2.2. Staining Assay and FACS

**Figure 7:**
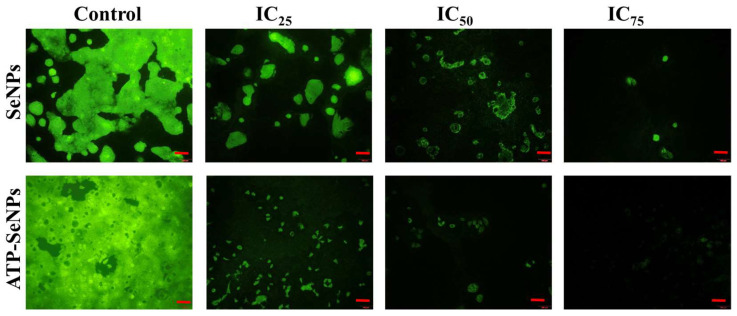
Analysis of SeNPs and ATP-SeNPs-induced mitochondrial membrane potential loss in human colon cancer cell line by rhodamine 123 (Rh123) staining assay. Scale bar (100 µm).

**Figure 8:**
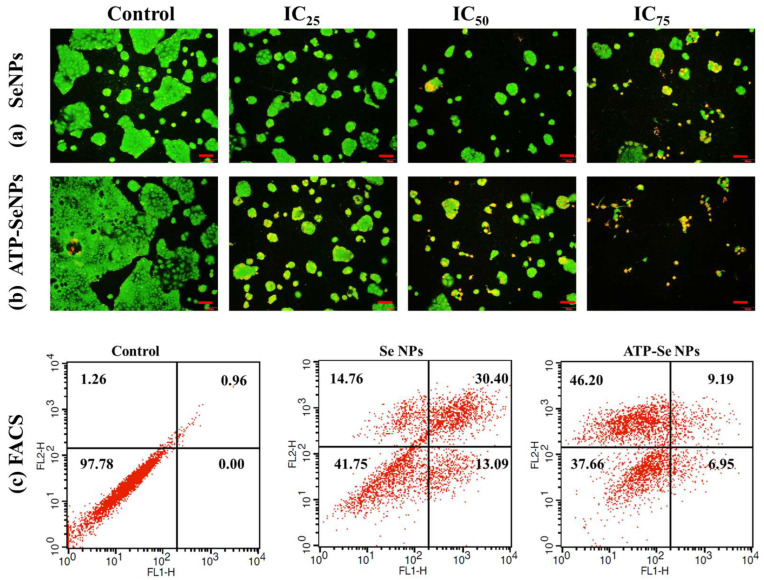
Analysis of SeNPs (**a**) and ATP-SeNPs (**b**) induced cell death stages in human colon cancer (caco-2) cell line by AO/EB staining assay. Flow cytometer analysis (FACS) of SeNPs and ATP-SeNPs induced apoptosis in human colon cancer (caco-2) cell line by Annexin V-FITC-PI staining (**c**). FL1 (Annexin V-FITC) and FL2 (PI). Scale bar (100 µm).

DAPI staining assay is commonly performed to observe any toxicant-induced nucleus damage in mammalian cells. The DAPI stain can effectively bind to the fragmented or crescent structure of the nucleus/dead cells compared to the healthy cell nucleus [46,47]. Similarly, the current study found dose-dependent cytotoxicity in the caco-2 cell line, but higher fragmented or crescent cells were found at the IC_75_ concentration of ATP-SeNPs-treated cells (Figure 6c). The Rh123 staining assay was then used to compare the effects of SeNPs and ATP-SeNPs on the induction of mitochondrial membrane potential (MMP) loss in the caco-2 cell line (Figure 7). Although both nanoparticles triggered the MMP loss in the caco-2 cell line, the higher MMP loss was found with the cells treated with ATP-SeNPs compared to SeNPs (Figure 7). Following this, the AO/EB staining assay was employed to observe the apoptosis stages in the caco-2 cells treated with either SeNPs or ATP-SeNPs (Figure 8a,b). The number of early apoptosis, apoptosis, and necrosis cells increased as the dose of both nanoparticles increased, but the high concentration of ATP-SeNPs exhibited significant necrosis and cell washout due to toxicity-induced floating of the dead cells. FACS was used to determine the apoptosis stages in SeNPs or ATP-SeNPs treated caco-2 cells using the Annexin FITC/PI staining kit (Figure 8c). The results demonstrated that the untreated control group exhibited 97.78% healthy cells, 0.00% early apoptosis cells, 0.96% late apoptosis cells, and 1.26% necrosis cells. SeNPs treatment resulted in 41.75% healthy cells, 13.09% early apoptosis cells, 30.40% apoptosis cells, and 14.76% necrosis cells. The case of the ATP-SeNPs showed 37.66% live cells, 6.95% early apoptosis, 9.19% late apoptosis, and 46.20% necrosis cells (Figure 8c). These results indicated that the SeNPs and ATP-SeNPs did not show significant changes in total cell death. The necrosis was found to be higher in the ATP-SeNPs (46.20%) compared to SeNPs (14.76%) (Figure 8c), which indicated the successful penetration of the ATP-SeNPs in caco-2 cells and resulting purinoceptor-targeted cell death in the human colon cancer cells (caco-2 cells) [3,20,21].

#### 2.2.3. Hemolysis and Egg CAM Toxicity

**Figure 9:**
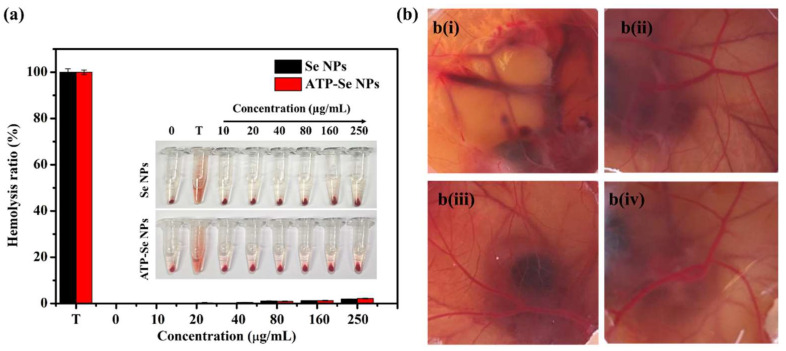
Hemolysis activity of ATP-SeNPs, Triton X-100 (T); insert image is of hemolysis observation image (**a**). Chick chorioallantoic membrane assay (**b**). NaOH (1 M; **b i**), PBS (**b ii**), SeNPs (**b iii**), and ATP-SeNPs (**b iv**).

The nanoparticles’ intravenous injection can form a protein corona due to the blood protein absorption [48]. Moreover, the blood compatibility of the nanoparticles is essential for the intravenous injection of the developed nanoparticles, and it is essential to study the blood and nanoparticle interactions [49]. Thus, the hemolytic property of the SeNPs and ATP-SeNPs was determined in RBC. Both nanoparticles were found to be biocompatible with RBC (Figure 9a). Similarly, earlier studies have reported the hemolytic properties of various nanoparticles such as iron oxide, carbon nanotubes, silver, silica, and gold nanoparticles [50,51,52,53,54]. These results indicate that the hemolysis properties of the nanoparticles varied based on the surface coating, type, size, nature, and surface charge [55]. Then, the egg CAM assay was performed to further confirm the biocompatibility of these nanoparticles in in vivo chick chorioallantonic membrane (CAM) toxicity assay. As seen from Figure 9b, the treatment of NaOH (1 M) caused significant blood vein damage, hemorrhage, and blood precipitation, but the PBS, SeNPs, and ATP-SeNPs treatments did not show any significant indications of egg CAM toxicity (Figure 9b).

## 3. Materials and Methods

### 3.1. Chemicals, Microorganisms, and Cell Lines

Sodium selenite, Adinosine 5′-triphosphate disodium salt hydrate (ATP), rhodamine 123 (Rh123), 4′,6-diamidino-2-phenylindole (DAPI), acridine orange (AO), and ethidium bromide (EB) were purchased from Sigma-Aldrich, Burlington, MA, USA. The cell viability assay kit (CELLO MAXTM) was procured from MediFab, Seoul, Korea. Gibco Dulbecco’s Modified Eagle Medium (DMEM), fetal bovine serum, penicillin, and streptomycin were bought from ThermoFisher Scientific, Waltham, MA, USA. Potato Dextrose Broth (PDB) and agar were obtained from the MB cell, Seoul, and Daejung Chemicals & Metals Co. Ltd., Siheung-si, Gyeonggi-do, Korea, respectively. Human embryonic kidney cells (HEK293 cell line) and human colon cancer cells (caco-2 cell line) were purchased from the Korean Cell Line Bank, (KCLB), Seoul, Korea. The previously reported *T. harzianum* (SKCGW012) strain was used for the synthesis of SeNPs [56].

### 3.2. Preparation of Trichoderma Extract and Synthesis of SeNPs and ATP-SeNPs

Three plugs (9 mm) of actively growing *T. harzianum* (SKCGW012) strain were inoculated in 100 mL of sterile PDB in a 200 mL Erlenmeyer flask and incubated for 5 days at 25 ± 2 °C. The mycelium of the strain was collected by filtration through a muslin cloth. The mycelium was washed with distilled water to remove the residual media and other impurities. Then, 20 g of mycelium was dissolved in sterile distilled water and sonicated for 5 min then kept in a shaking incubator overnight at 25 ± 2 °C for extraction. Afterward, the extract was collected by filtration through a Whatman No.1 filter paper and labeled as TE. The 10 mL of TE (3 mg/mL) was added to 90 mL of sterile distilled water containing 25 mM of sodium selenite. Then, the reaction mixture was kept in a shaking incubator at 28 ± 2 °C and 180 rpm for 120 h. The formation of the nanoparticles was primarily confirmed by color changes in the reaction mixture. Following this, the aliquots of the samples were collected at different time intervals (0–120 h) and measured using UV–Vis spectrophotometry scan, ranging from 200–500 nm. After the reaction period, the nanoparticles were collected by a two-phase centrifugation process at 4000 rpm for the removal of unreacted bounded materials for 10 min and at 18,000 rpm for 20 min for the collection of nanoparticles. In addition, the final product of the SeNPs was decorated with ATP and dialyzed against sterile distilled water at 4 °C overnight to remove the unbounded SeNPs according to methods reported earlier [3].

### 3.3. Characterization of SeNPs and ATP-SeNPs

The physicochemical and morphological properties of SeNPs and ATP-SeNPs were characterized by various material characterization techniques. The synthesis of the SeNPs was first analyzed by the UV–Vis spectrophotometer (Biochrom Ltd. Libra S80, Cambridge, UK). The crystallinity size was determined through X-ray diffraction (XRD; XRD, X’pert-pro MPD-PANalytical, Almelo, The Netherland) analysis. The morphology and size of the nanoparticles were observed by field emission transmission electron microscopy (FE-TEM; JEOL (JEM-2100F), Peabody, MA, USA). The elemental analysis was performed by energy-dispersive X-ray spectroscopy (EDS; JEOL (JEM-2100F), Peabody, MA, USA). The hydrodynamic particle size and zeta potential of the nanoparticles were measured using the Zeta Potential Particle Size Analyzer (Malvern (ZSP), Cambridge, UK). The chemical functional changes in the SeNPs and ATP decorated SeNPs were analyzed by the FTIR spectrophotometer (PerkinElmer (Frontier), Waltham, MA, USA).

### 3.4. Cytotoxicity Assay

The cytotoxicity of the SeNPs and ATP-SeNPs was tested on two model cell lines, including the HEK293 cell line and the caco-2 cell line, using the water-soluble tetrazolium salt (WST-1) cell proliferation assay. In brief, the HEK293 cell line and the caco-2 cell line were cultured for 24 h at 37 °C in humidified 5% CO_2_ environment in DMEM media containing 5% FBS and 1% P&S. After that, the cells were collected through the trypsinization process using the EDTA-trypsin, then washed with cold PBS, and the concentration of cells was adjusted to 1 × 10^4^ by dissolving in DMEM media. A total of 100 µL of cells (1 × 10^4^) was seeded in the 96-well plates and allowed to grow in the above-mentioned CO_2_ environment until reaching the cell confluence of 80–90%. Then, 10 µL of different concentrations of SeNPs and ATP-SeNPs (0, 20, 40, 80, 160, 250 µg/mL) were added to each well and incubated for 16 h in the above-mentioned conditions. After the incubation, 10 µL of premix of the WST-1 was added to each well and incubated for 90 min at 37 °C in a humidified 5% CO_2_ environment. Following this, the optical density was measured at 570 nm by a UV–Vis spectrophotometer (Biochrom Ltd. Libra S80, Cambridge, UK). The cell viability was calculated using the standard protocols described earlier [57]. Following this, several separate experiments were performed for further determination of cytotoxicity using the cell stains, including the DAPI staining assay, for nucleus damage, and Rh123 for ΔΨm loss. AO/EB staining assay for determination of apoptosis, and Annexin FITC-PI staining assay of FACS for measurement of cell death stages using the methods reported elsewhere [58,59]. The cellular uptake of the SeNPs was determined by ICP [3].

### 3.5. Hemolysis Assay

Hemolytic activity of the SeNPs and ATP-SeNPs was tested on the red blood cells (RBC). The hemolysis assay was performed in sheep blood (Carlina, Korea) by applying the method described earlier [60]. For the RBC preparation, 10 mL of PBS was added to 1 mL of blood and mixed well, then centrifuged at 2000 rpm for 15 min at 4 °C. The collected RBC was washed with PBS three times. For the assay, 200 µL of RBC (4% *v*/*v*) and 400 µL of various nanoparticles (0–250 μg/mL) were mixed and kept in an incubator for 45 min at 37 °C. Then the reaction mixture was centrifuged at 2000 rpm for 15 min at 4 °C and the optical density of the supernatant was measured at 545 nm using the UV–Vis spectrophotometer (Biochrom Ltd. Libra S80, Cambridge, UK). Here, the Triton X-100 (1% (*v*/*v*)) and PBS were considered as positive and blank controls, respectively. Hemolysis (%) = (test/control) × 100.

### 3.6. Chick Egg CAM Assay

The effect of SeNPs and ATP-SeNPs on chick embryos was analyzed through an in vivo chick embryo chorioallantoic membrane (CAM) assay [61]. The fertile eggs were incubated at 37 ℃ with 60% humidity for a week. Then, the egg surface was sterilized with 70% ethanol and the eggshell was carefully opened without damaging the CAM. Then, 200 μL of 200 μg/mL of each nanoparticle were gently added to the egg CAM and incubated at ambient temperature for 5 min. Here, NaOH (1 M) and PBS were used as positive and negative controls, respectively. In the samples, induced embryonic changes (blood vessel damage and hemorrhage) were noted according to the indicators described in the earlier work [62].

### 3.7. Statistical Analysis

The experiments were repeated three times independently, and the mean and standard error (SE) were calculated by descriptive statistics. The significance between the samples and concentration was then analyzed by ANOVA using the IBM SPSS statistics. The graphs were generated with OriginPro 8.5.

## 4. Conclusions

SeNPs are reportedly a novel agent with promising anticancer activity with less adverse effects, but their activity is limited due to their limited cellular internalization and uptake. To overcome this drawback and improve the anticancer activity of SeNPs through enhancing the cellular uptake, the present work synthesized the SeNPs using TE and conjugated them with ATP to enhance their cellular permeabilization and cancer cell-targeted cytotoxicity in caco-2 cell line. ATP is known to specifically target the overexpressed P2 (purinoceptors) receptor in cancer cells. Both nanoparticles were sized <200 nm and were spherical in structure, with an amicable zeta potential and PDI for biomedical application. FTIR results indicated the role of compounds in the TE as a reducing capping agent for the syntheses of SeNPs. In addition, the FTIR spectrum and DLS revealed that SeNPs and ATP established a stable conjugation through the Se–NH conjugation and a covalent bond through electrostatic interaction. Overall, the characterization results proved the successful formation of the ATP-SeNPs. The ATP-SeNPs exhibited higher cytotoxicity in caco-2 cell line through nucleus damage and MMP loss. Our results demonstrate a novel approach for improving the anticancer activity of the SeNPs through targeting the P2 receptors in the caco-2 cells. However, further evaluation is required in human cancer animal model studies to develop the ATP-SeNPs as a candidate drug for cancer-targeted therapeutics.

## Data Availability

Data is contained within the article.
